# Cape Feather Coloration Signals Different Genotypes of the Most Polymorphic MHC Locus in Male Golden Pheasants (*Chrysolophus pictus*)

**DOI:** 10.3390/ani11020276

**Published:** 2021-01-22

**Authors:** Hong-Yi Liu, Ke He, Yun-Fa Ge, Qiu-Hong Wan, Sheng-Guo Fang

**Affiliations:** 1 MOE Key Laboratory of Biosystems Homeostasis & Protection, State Conservation Centre for Gene Resources of Endangered Wildlife, College of Life Sciences, Zhejiang University, Hangzhou 310058, China; hongyi_liu@njfu.edu.cn (H.-Y.L.); heke@zafu.edu.cn (K.H.); yunfa_ge@163.com (Y.-F.G.); qiuhongwan_2013@163.com (Q.-H.W.); 2 College of Biology and the Environment, Nanjing Forestry University, Nanjing 210037, China

**Keywords:** feather coloration, major histocompatibility complex, male quality, golden pheasant

## Abstract

**Simple Summary:**

Ornamental feather coloration in birds is deemed an honest signal of male quality, but is seldom embodied at the functional genetic level. The major histocompatibility complex is involved in the immune response, thereby affecting an organism’s fitness. Here, we tested the hypothesis that the coloration of the cape feather signals genetic quality in the male golden pheasant. We measured brightness, chroma, and hue as the main parameters to quantify color differences. Then, we investigated the genotypes of all major histocompatibility complex loci associated with adaptive immunity. The results showed that heterozygosity of the most polymorphic major histocompatibility complex locus was positively related with the brightness and chroma of cape feathers. Our study suggested that cape feather coloration might signal different genotypes of the most polymorphic major histocompatibility complex locus in male golden pheasants.

**Abstract:**

Ornamental feather coloration is usually a reflection of male quality and plays an important role during courtship, whereas the essence of male quality at the genetic level is not well understood. Major histocompatibility complex (MHC)-based mate choice has been observed in various vertebrates. Here, we investigated the relationship between the coloration of cape feathers and the MHC genotypes in golden pheasants (*Chrysolophus pictus*). We found that feather coloration differed sharply among different individuals (brightness: 1827.20 ± 759.43, chroma: 1241.90 ± 468.21, hue: 0.46 ± 0.06). Heterozygous individuals at the most polymorphic MHC locus (IA2) had brighter feathers than homozygous individuals (Z = −2.853, *p* = 0.004) and were more saturated in color (Z = −2.853, *p* = 0.004). However, feather coloration was not related to other MHC loci or to overall genetic heterozygosity (*p* > 0.050). Our study suggested that coloration of cape feathers might signal IA2 genotypes in golden pheasants.

## 1. Introduction

Ornamental feather coloration is deemed an honest signal of male quality and plays a key role in avian courtship [1,2,3,4,5,6,7]. Many studies have demonstrated that differences in the brightness, chroma, and hue of feather coloration reflect male quality, and thus affect mating success [6,7]. In correlational studies, male quality has usually been indicated by physiological indices (such as the immunoglobulin level and number of parasites in the host) and habitat quality (such as the size of the habitat and quantity of food in the habitat) [2,3,7]. Male quality is seldom embodied at the genetic level.

The major histocompatibility complex (MHC) is a highly polymorphic gene family, which encodes glycoprotein receptors that are responsible for presenting antigens to the immune system to initiate an adaptive immune response [8]. The heterozygote advantage hypothesis suggests that heterozygous genotypes have a higher relative fitness than homozygous genotypes. The diversity mechanism of MHC is a classic example of heterozygote advantage selection, as MHC heterozygous genotypes can potentially provide resistance to a wider range of antigens [9,10]. Many studies have found direct evidence for MHC-based mate choice [4,6,11,12,13]. The MHC genotype might be a good candidate index to reflect the variation of male quality [4,6]. The relationship of the MHC genotype with birdsong and preen oil has been well studied in songbirds and seabirds [14,15,16]. To date, only a few studies have attempted to establish the essential relationship between the MHC genotype and ornamental feathers [4,6,17,18]. Besides these studies, there are few that have investigated the relationship between the MHC genotype and brightness, chroma, and hue of ornamental feather coloration.

**Figure 1:**
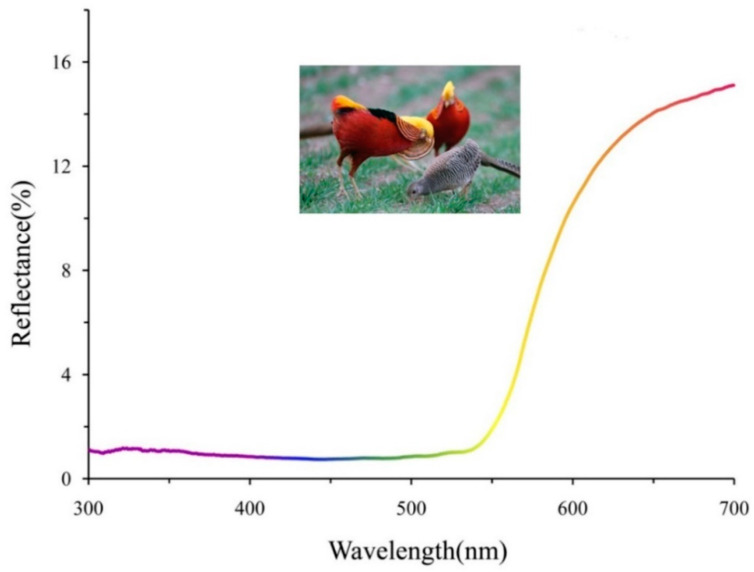
Reflectance of cape feathers from male golden pheasant. The range of bird visual sensitivity is 300–700 nm. The curve was represented by the average value of all individuals. The photograph of the golden pheasant was provided by Mr. Ming-yun Zhang.

The golden pheasant (*Chrysolophus pictus*) is a sexual dichromatic bird that is an endemic species of China (Figure 1). Male birds possess multicolored feathers that are absent in females. We speculated that the orange and black cape feather could reflect genetic quality due to its dramatic function in courtship displays (Figure 1) [19,20]. Here, we tested the hypothesis that the coloration of the cape feather signals genetic quality in the male golden pheasant. We adopted brightness, chroma, and hue as the main parameters to quantify color differences. Then, we investigated the genotypes of five MHC loci for each individual. In addition, we investigated whether the overall genetic heterozygosity (H_o_) influenced coloration of the cape feather using simple sequence repeat (SSR) markers. Finally, we explored the relationship between ornamental feather coloration and genetic quality by comparing the color differences between heterozygous and homozygous genotypes.

## 2. Materials and Methods

The experimental population was semi-captive and consisted of 48 males. We collected a cape feather from each individual to use for coloration quantification. We chose the outer golden layer for analysis due to its large size and foreground. Reflectance was measured using an AvaSpec-2048 spectrometer and AvaLight-DH-CAL light source. We anchored the probe perpendicular to the feathers and set the exposure time at 100 ms. Then, we measured the reflectance using an AvaSoft 7.3 (Avantes, Apeldoom, the Netherlands). The measured values covered all the reflectance data of the avian visible spectrum (300–700 nm).

Genomic DNA was extracted from blood samples using the phenol-chloroform method. Previously, we have characterized the MHC class I genes (IA1 and IA2) and class II genes (IIB1, IIB2, and IIB3) in golden pheasants [8]. We used locus-specific primers to amplify polymorphic exon 2 of all MHC loci and identified the genotypes of each individual with sequencing technology and single-strand conformation polymorphism-heteroduplex analysis. Locus-specific primers were provided from two studies [21,22] (Table S1). Meanwhile, we measured the H_o_ of each individual using 12 SSR markers developed in this species [23] (Table S2). PCR amplification, sequencing, and genotyping were performed following our previously published methods [21,22,23] (Table S3).

The brightness, chroma, and hue of feather coloration were analyzed based on the reflectance data. The values were calculated using the following spectral ranges: UV 300–400 nm; blue 400–475 nm; green 475–550 nm; yellow 550–625 nm; red 625–700 nm. Brightness was calculated as the sum of relative reflectance across the entire spectral range of 300–700 nm. Chroma was defined as the square root of (R_625–700_ − R_475–550_)^2^ + (R_550–625_ − R_400–475_)^2^. Hue equaled the value of the following formula: Sin[(R_550–625_ − R_400–475_)/chroma]. All calculation formulas have been described by Endler [24]. Outliers were detected by boxplots in SPSS 16.0 [25]. H_o_ was calculated as the proportion of heterozygous loci among the 12 SSR markers [26]. Individuals were divided into two groups on the basis of their H_o_ values (if an individual’s H_o_ was more than the average H_o_ of all individuals, it was categorized as heterozygous). The coloration difference between heterozygous and homozygous individuals was compared using the nonparametric Mann–Whitney U tests with *p* values from Monte Carlo procedures in SPSS 16.0 [25].

## 3. Results

The reflectance of cape feathers varied greatly within the wavelength spectrum (Figure 1). The reflectance was very low within the range of 300–550 nm, especially in the blue wavelength. High reflectance existed in long wavelength regions, as the inflection point of reflectance emerged in the yellow wavelength (Figure 1). By calculating and comparing, the brightness, chroma, and hue of the feather coloration differed sharply between individuals. The brightness ranged from 718.91 to 3918.62, and the average value was 1827.20 ± 759.43 (Table 1). The maximum, minimum, and average values of the chroma were 2484.95, 536.43, and 1241.90 ± 468.21, respectively (Table 1). The average value of the hue was 0.46 ± 0.06, with a range from 0.31 to 0.55 (Table 1).

Many MHC and SSR sequences were isolated from the 48 individuals. For the MHC loci, there were 15 alleles at the most polymorphic locus (IA2) (Table 2). Even for the least polymorphic locus, there were still five alleles isolated (IIB2) (Table 2). The number of heterozygous individuals was greater than that of homozygous individuals at most MHC loci (Table 2). For the SSR markers, as many as 18 alleles were detected at the most polymorphic locus, whereas only two alleles existed at the least polymorphic locus (Table 2). The average H_o_ was 0.76 and only one individual had a H_o_ that was below 0.5, which indicated that the number of heterozygous males was greater than that of homozygous males at most SSR loci.

**Figure 2:**
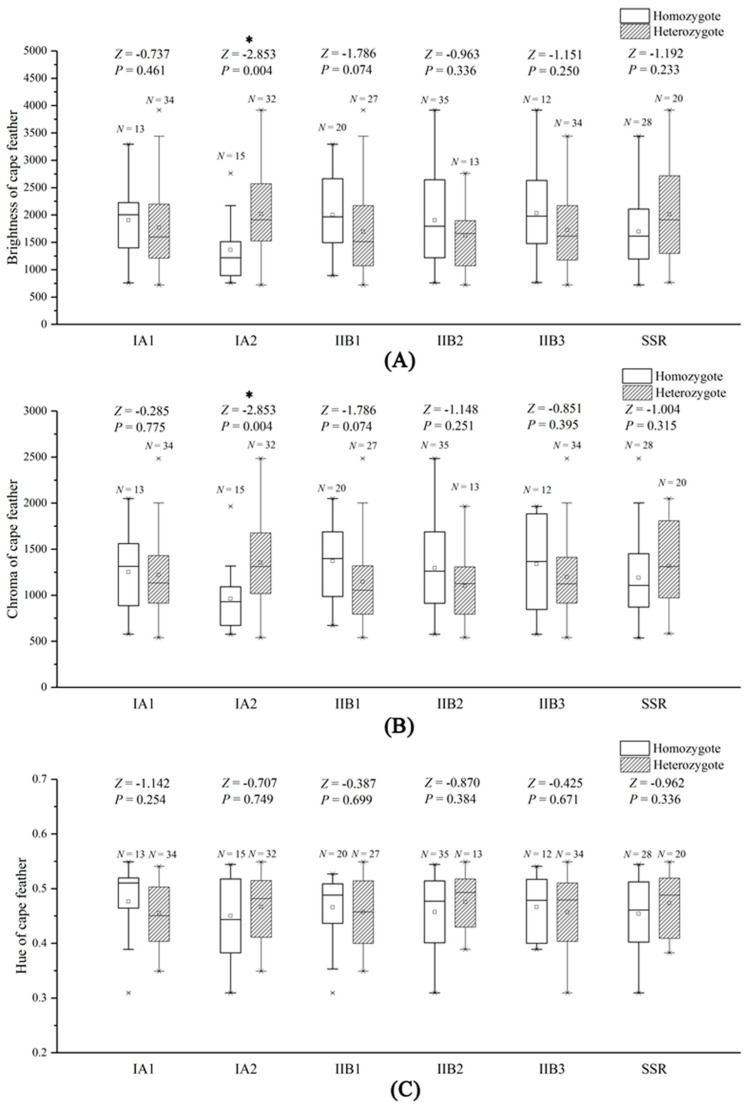
Relationships between coloration ((**A**) brightness, (**B**) chroma, (**C**) hue) of cape feathers and heterozygosity. Heterozygosity of the IA2 locus was positively related to the brightness and chroma of cape feathers (*p* < 0.005), whereas the heterozygosity of other MHC loci and H_o_ was unrelated to the coloration of cape feathers (*p* > 0.050). * means significant difference.

For the relation between feather coloration and MHC genotypes, individuals that were heterozygous at the IA2 locus had brighter cape feathers (*Z* = −2.853, *p* = 0.004, Figure 2A). The feathers from the same heterozygous individuals were also more saturated in color (higher chroma) than the homozygous individuals (*Z* = −2.853, *p* = 0.004, Figure 2B). However, the genotypes of the other MHC loci were unrelated to the coloration of cape feathers (*p* > 0.050, Figure 2A–C). For the relation between feather coloration and H_o_, neither the brightness, chroma, nor hue could express the difference in H_o_ values (*p* > 0.050, Figure 2A–C).

## 4. Discussion

Ornamental feather coloration is usually considered an honest signal of male quality [1,2,3,4,5,6,7]. However, in most cases, little is known about the variation of male quality at the genetic level. Based on the present study in the golden pheasant, we found that coloration of the cape feather was indicated by genotype differences in the IA2 locus. The feathers from heterozygous individuals were brighter and more saturated in color than feathers from homozygous individuals (Figure 2A,B). Yet, there was no evidence that the genotypes of other MHC loci were related to the coloration of cape feathers (Figure 2A–C). These results might be related to the roles of different MHC loci. Important MHC loci are usually presented as being highly polymorphic, as they may be subject to strong natural selection involved in variable antigens [10,21,22]. The variation in the IA2 locus might be very important for the golden pheasant. However, since there was no information on mate choice and offspring fitness, the significance of the IA2 locus could not be adequately identified in the present study. As reported in *Geothlypis trichas* and *Nipponia nippon*, the feather is a key trait of mate choice for picky females, and this supports the Hamilton–Zuk hypothesis [6,18,27]. The relationship between the IA2 locus and feather coloration suggests the genotypes of this locus may affect mate choice during mating season, which is worth considering in the next step. Furthermore, we also investigated whether the H_o_ value affected ornamental feather coloration. No relationship between H_o_ and feather coloration was identified, which might be caused by the nature and/or the limited number of SSR loci used. Conversely, studies in lizards showed that male throat coloration may act as a signal of MHC genotype to conspecifics, whereas H_o_ may contribute to mating success [26,28].

Significant differences in the coloration of cape feathers between different individuals under the same circumstances could reveal genetic quality and demonstrate the concept of honest advertisement in golden pheasant (Figure 1 and Figure 2A,B). However, several studies have demonstrated that the relationship between the MHC genotype and phenotypic characteristic may vary among different species [14,15,16,17,18]. In addition to the optical parameters, the area, shape, and location of ornamental feather coloration might also transmit other information on male quality [3]. Thus, the relationship between ornamental feathers, male quality, and mate choice in sexual dichromatic birds is still an interesting aspect that requires further studies.

## 5. Conclusions

The heterozygosity of the most polymorphic IA2 locus was positively related with the brightness and chroma of cape feathers. The relationship among MHC genotype, phenotypic characteristic, and mate choice in golden pheasant needs to be verified in further studies.

## Figures and Tables

**Table 1:** Brightness, chroma, and hue of the feather coloration in golden pheasant.

Parameter	Minimum	Maximum	Average ± SD
Brightness	718.91	3918.62	1827.20 ± 759.43
Chroma	536.43	2484.95	1241.90 ± 468.21
Hue	0.31	0.55	0.46 ± 0.06

**Table 2:** Summary of the number of alleles and percentage of heterozygotes at MHC and SSR loci.

	MHC Class I		MHC Class II			SSR		
	IA1	IA2	IIB1	IIB2	IIB3	Minimum	Maximum	Average
Number of alleles	8	15	10	5	6	2	18	10.42
Percentage of heterozygote (%)	72.3	68.1	57.4	27.1	73.9	39.5	97.9	75.3

## Supplementary Materials

The following are available online at https://www.mdpi.com/2076-2615/11/2/276/s1.
